# Comparing Detection Schemes for Adversarial Images against Deep Learning Models for Cancer Imaging

**DOI:** 10.3390/cancers15051548

**Published:** 2023-03-01

**Authors:** Marina Z. Joel, Arman Avesta, Daniel X. Yang, Jian-Ge Zhou, Antonio Omuro, Roy S. Herbst, Harlan M. Krumholz, Sanjay Aneja

**Affiliations:** 1 Department of Dermatology, Johns Hopkins University School of Medicine, Baltimore, MD 21205, USA; 2 Department of Therapeutic Radiology, Yale School of Medicine, New Haven, CT 06510, USA; 3 Department of Chemistry, Physics and Atmospheric Science, Jackson State University, Jackson, MS 39217, USA; 4 Department of Neurology, Yale School of Medicine, New Haven, CT 06510, USA; 5 Department of Medicine, Yale School of Medicine, New Haven, CT 06510, USA; 6 Center for Outcomes Research and Evaluation (CORE), Yale School of Medicine, New Haven, CT 06510, USA

**Keywords:** artificial intelligence, deep learning, cancer classification, medical imaging

## Abstract

**Simple Summary:**

While deep learning has become a powerful tool in analysis of cancer imaging, deep learning models have potential vulnerabilities that pose security threats in the setting of clinical implementation. One weakness of deep learning models is that they can be deceived by adversarial images, which are manipulated images that have pixels intentionally perturbed to alter the output of the deep learning model. Recent research has shown that adversarial detection models can differentiate adversarial images from normal images to protect deep learning models from attack. We compared the effectiveness of different adversarial detection schemes, using three cancer imaging datasets (computed tomography, mammography, and magnetic resonance imaging). We found that that the detection schemes demonstrate strong performance overall but exhibit limited efficacy in detecting a subset of adversarial images. We believe our findings provide a useful basis in the application of adversarial defenses to deep learning models for medical images in oncology.

**Abstract:**

Deep learning (DL) models have demonstrated state-of-the-art performance in the classification of diagnostic imaging in oncology. However, DL models for medical images can be compromised by adversarial images, where pixel values of input images are manipulated to deceive the DL model. To address this limitation, our study investigates the detectability of adversarial images in oncology using multiple detection schemes. Experiments were conducted on thoracic computed tomography (CT) scans, mammography, and brain magnetic resonance imaging (MRI). For each dataset we trained a convolutional neural network to classify the presence or absence of malignancy. We trained five DL and machine learning (ML)-based detection models and tested their performance in detecting adversarial images. Adversarial images generated using projected gradient descent (PGD) with a perturbation size of 0.004 were detected by the ResNet detection model with an accuracy of 100% for CT, 100% for mammogram, and 90.0% for MRI. Overall, adversarial images were detected with high accuracy in settings where adversarial perturbation was above set thresholds. Adversarial detection should be considered alongside adversarial training as a defense technique to protect DL models for cancer imaging classification from the threat of adversarial images.

## 1. Introduction

Diagnostic imaging is a cornerstone of clinical oncology with an increasingly important role in cancer detection, treatment planning, and response assessment. With the increasing use of various diagnostic imaging modalities for cancer management, there has been a growing desire to leverage machine learning (ML) methods to improve diagnostic image analysis [1]. Deep learning (DL) models in particular have shown significant promise in helping interpret various diagnostic imaging modalities such as computed tomography (CT), magnetic resonance imaging (MRI), and X-ray images across cancer types [2,3,4,5]. Recently, the US Food & Drug Administration (FDA) has approved multiple DL-based computer vision algorithms for medical imaging to be used by healthcare start-ups [6,7,8]. Recent examples are FDA-approved DL algorithms for breast cancer screening (e.g., Transpara and Mammoscreen) using digital mammography and digital breast tomosynthesis [8]. As these DL-based medical imaging systems are already approved for medical diagnosis without clinician input, DL models for medical imaging have imminent potential to be utilized in real-world cancer diagnostics. Some of the incentives for using DL models in clinical diagnostics to supplement or even replace human decision making include mitigating healthcare costs as well as human error [9].

Despite the success of DL models across various imaging tasks, there remain notable vulnerabilities which may hinder clinical its implementation. Specifically, DL models are vulnerable to adversarial images—images engineered with slight perturbations to cause DL models to give false predictions. The weakness of DL models against adversarial images stems from the fact that DL models are algorithmically unstable, producing significantly different outputs when the given inputs are subtly modified [10,11]. Clinically, this could lead to a misdiagnosis of non-cancerous lesions as cancerous, or worse, miss potential cancers present in diagnostic images. Although adversarial images are often difficult to distinguish visually from clean images, they have been shown to significantly decrease DL model classification accuracy [12,13,14]. Previously, it was thought that limiting access to training data—medical images for classification—to be publicly unavailable would prevent the security threat of adversarial images, as generation methods for adversarial perturbations usually require the use of original training data. However, Minagi et al. showed that transfer learning from non-medical images can be used to generate adversarial perturbations for medical images without using actual medical images as training data [15]. Thus, bad actors can potentially create adversarial images to deceive medical DL models and manipulate clinical decision making even without access to the medical images used for training, presenting opportunities for healthcare fraud and risks to patient safety [9,15]. For example, adversarial images could be used to distort patient diagnosis to generate false referrals or inappropriate treatments or medication prescriptions [16]. In light of these potential threats to the healthcare system from the manipulation of DL models, solely relying on DL algorithms to automate medical imaging tasks without human intervention can be dangerous and irresponsible despite its cost effectiveness. 

Although adversarial training methods have been developed to create robust DL models which are more successful at classifying adversarial images, they have shown limited efficacy on oncologic images, and their improvement of model robustness against adversarial images comes at a tradeoff of decreasing their standard accuracy against clean images [17,18,19]. Furthermore, adversarial training is very computationally expensive as an iterative fine-tuning method [20]. An alternative solution to mitigate misclassification of adversarial images is developing methods which identify adversarial images before a DL model makes a prediction. 

In this study, we investigate the efficacy of five different methods using DL- and ML-based detection models to classify adversarial images across three oncologic imaging modalities: CT, mammography, and MRI. Additionally, we examine the utility of combining adversarial image detection with adversarial training methods to improve DL model robustness. 

## 2. Materials and Methods

### 2.1. Ethics Declaration

Research was conducted in accordance with the Declaration of Helsinki guidelines and approved by the Yale University Institutional Review Board (Protocol ID: HIC#2000027592). Informed consent was obtained from all participants in this study.

### 2.2. Datasets

Experiments were conducted on three datasets of different imaging modalities: CT, mammography, and MRI. We used CT imaging data composed of 1018 thoracic CT scans and 2600 lung nodules from the Lung Image Database Consortium and Image Database Resource Initiative (LIDC-IDRI) collection [21]. Thoracic radiologists identified the lung nodules used for the DL model, and associated pathologic reports were used to determine the presence of malignancy. Radiologist consensus was used to determine malignancy for patients without a pathologic determination. 

We used mammography imaging data consisting of 1696 lesions from 1566 patients from the Curated Breast Imaging Subset of Digital Database for Screening Mammography (CBIS-DDSM) [22]. Regions of interest were algorithmically derived using clinical metadata and were used to determine mammographic lesions. Verified pathologic reports were used to determine the presence of malignancy.

We used brain MRI data from 831 patients from a single institution brain metastases registry [1]. A multi-disciplinary team of radiation oncologists, neurosurgeons, and radiologists identified regions of interest. For 4000 brain lesions that we identified, we determined the presence of malignancy based on pathologic confirmation or clinical consensus.

### 2.3. Models

The classification models had a VGG16 convolutional neural network architecture with pretrained weights [18,23]. We used data augmentation—horizontal and vertical flips, and random rotations—to train the classification models and optimized the models using stochastic gradient descent. DL classification models were fixed post training and used for adversarial detection experiments. Each model was trained to classify the presence or absence of a malignancy in an image. Each imaging dataset was divided into a training set and a validation set using a ratio of 2:1. For image processing, each image was center cropped, resized, and normalized. Classes were balanced for each dataset. 

For adversarial detection, we used five different detection models. Two were ImageNet-pretrained convolutional neural networks with ResNet50 and DenseNet-121 architecture, respectively. We also used a DenseNet-121 model to extract deep features from images and separately used logistic regression (LR), random forest (RF), and support vector machine (SVM) as the detection classifiers based on the extracted deep features. Each detection model was trained on the combination of the original training set and adversarial images generated from the training set, and tested on the combination of the original test set and adversarial images generated from the test set. 

Details regarding model architecture and hyperparameter selection for model training are provided in the Supplementary Tables S1–S5. For both classification and detection models, model performance was evaluated using accuracy—the percentage of images for which the model was able to predict to correct label.

### 2.4. Adversarial Image Generation

**Figure 1:**
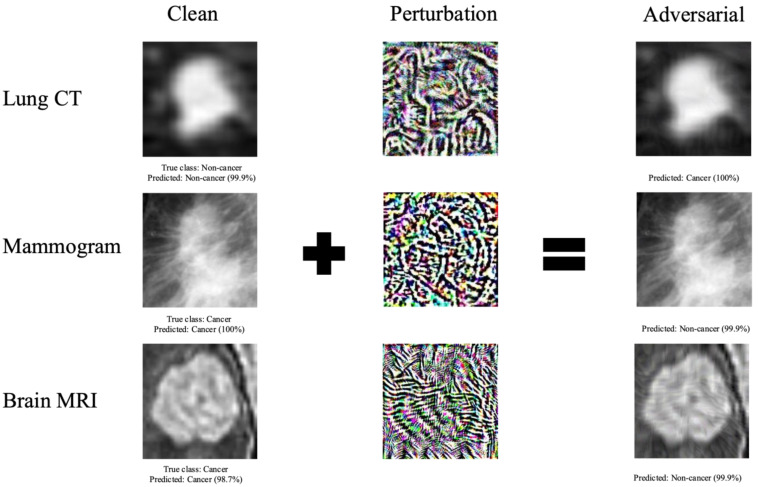
Examples of clean images, adversarial perturbations, and resulting adversarial images generated using PGD attack method. The percentage displayed represents the prediction confidence— the probability predicted by the DL model that the image is of a certain class. Adversarial perturbations cause a change in the DL model classification of the image.

We considered three first-order adversarial attack methods: Fast Gradient Sign Method (FGSM), Projected Gradient Descent (PGD), and Basic Iterative Method (BIM). Using these attack methods, we crafted adversarial images on the medical image datasets (Figure 1). All the attacks considered are bounded under a predefined perturbation size ε, which represents the maximum change to each input image pixel. 

The single-step FGSM attack perturbs the clean image by a fixed amount along the direction (sign) of the gradient of adversarial loss [24]:

$$x_{adv}=x+\varepsilon \;sign\left(\nabla_{x}\;J\left(x,\;y\right)\right)$$

where J represents the loss function, x represents the original input image, and y represents the ground-truth label of input image.

PGD iteratively perturbs the clean image for a number of T steps with smaller step sizes; after each iteration, the updated adversarial image is projected onto the ε-ball of x [14]:

$$x^{t}=\prod_{\epsilon }\left(x^{t-1}+\alpha \;sign\left(\nabla_{x}J\left(x^{t},\;y\right)\right)\right)$$

where α represents the step size, ∏ represents the projection function, and x^t^ is the adversarial image at the t-th step.

BIM is the iterative version of FGSM, essentially performing FGSM multiple times with a step size α. It also clips the pixel values of the updated adversarial image after each step into a permitted range [25].

$$x^{t}=Clip_{x,\;\epsilon }\{x^{t-1}+\alpha \;sign\left(\nabla_{x}\;J\left(x^{t},\;y\right)\right)\}$$

We evaluated the performance of our VGG16 classification models using FGSM, PGD, and BIM adversarial image generation methods across different levels of pixel perturbation. Relative model sensitivity to adversarial images was assessed by the amount of perturbation ε required for adversarial images to substantially decrease model accuracy. 

### 2.5. Adversarial Detection

We used the same VGG16 classification models as for above attack experiments. Each detector model was trained on the combination of the clean training set and corresponding adversarial training set generated by BIM attack. Detector model training hyperparameters are detailed in the Supplementary Table S6. For each classification task, we measure detection performance by reporting the classification accuracy of the detector model on the combination of the normal test set and the corresponding adversarial test set generated through FGSM, PGD, or BIM attack. To assess the detectability of adversarial examples, we report the detection accuracies for the detector models against all three types of attacks of varying perturbation sizes across the datasets. 

### 2.6. Comparison of Approaches on Improving Classification Accuracy

We compared the efficacy of adversarial detection, adversarial training, and the combination of adversarial detection and adversarial training on improving classification accuracy of the DL model. Each scheme was evaluated on the combination of a clean test set and the corresponding adversarial test set generated via BIM attack with a fixed perturbation size of 0.004. We first evaluated the baseline accuracy of the original DL model on the combined test set. We then evaluated the accuracy of the adversarially trained DL model on the combined test set. For adversarial training, a multi-step PGD adversarial training method was used where for each batch of training images, half were normal images and half were adversarial images. For adversarial detection, we first used the ResNet detector to exclude images detected as adversarial and then evaluated the accuracy of the original DL model on the remaining dataset; we adjusted the accuracy by accounting for clean images wrongly excluded by the detector by including that number in the denominator of accuracy calculation. For the combined adversarial detection and adversarial training approach, we repeated the previous scheme but used the adversarially trained model instead of the original DL model for final accuracy evaluation.

The code was implemented in Python 2.7, with DL models using the TensorFlow v.1.15.3 framework and ML models using the scikit-learn 1.2.0 package [26,27]. Adversarial images were generated with the Adversarial Robustness Toolbox v.1.4.1 [28].

### 2.7. Code Availability

The source code for implementation of this paper is available online at Github: https://github.com/Aneja-Lab-Yale/Aneja-Lab-Public-Adversarial-Detection (accessed on 1 February 2023).

## 3. Results

All three DL models for CT, mammogram, and brain MRI datasets were highly susceptible to adversarial attacks. Before the application of adversarial attacks, our DL models achieved baseline classification accuracies of 75.4% for CT, 76.4% for mammogram, and 92.4% for MRI. Adversarial images generated using PGD with a perturbation size of 0.004 resulted in dramatic decreases in performance: a DL model accuracy of 25.6% for CT, 23.9% for mammogram, and 7.65% for MRI. 

**Figure 2:**
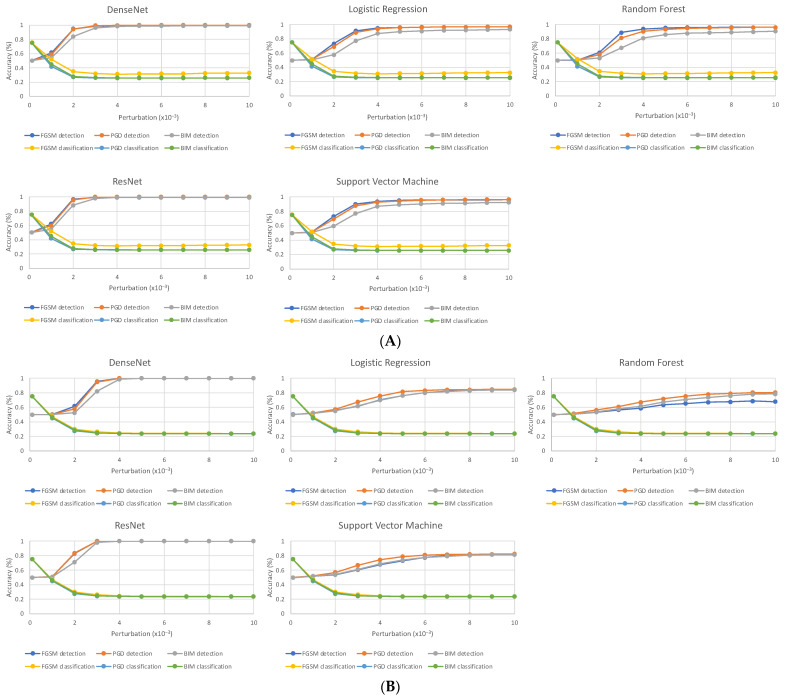

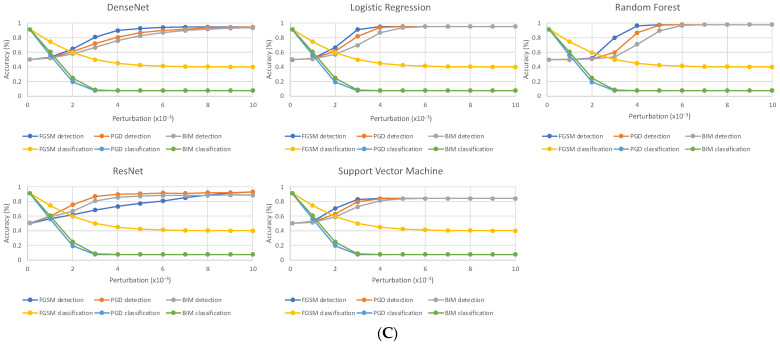
Detection accuracy (%) of detector models (DenseNet, Logistic Regression, Random Forest, ResNet, and Support Vector Machine) on the combination of normal and adversarial test samples along with classification accuracy (%) of VGG16 classification model on adversarial samples as L_∞_ maximum perturbation size ε is increased. As ε was increased, detection accuracy increased while classification model accuracy decreased for all datasets and attack types. Results are shown for (**A**) lung CT, (**B**) mammogram, and (**C**) MRI. Detection accuracy is measured as the percentage of images in the combined test set correctly classified by detection model to be normal or adversarial, while classification accuracy is measured as the percentage of images in the normal test set correctly classified by the VGG16 classification model as malignancy or no malignancy.

Our adversarial detection models showed strong performance for all attacks across all datasets for attacks of perturbation sizes larger than 0.004 (Figure 2, Table 1). In all cases, the detection accuracy increases as the maximum perturbation (ε) of the attack is increased. This is expected, as adversarial images with larger perturbation sizes are more easily distinguished from normal images due to greater differences in feature distribution. Adversarial images generated using PGD with a perturbation size of 0.004 were detected by ResNet detection model with an accuracy of 100% for CT, 100% for mammogram, and 90.0% for MRI, and were detected by the DenseNet detection model with an accuracy of 99.7% for CT, 99.9% for mammogram, and 80.5% for MRI. In contrast, the images were detected by the RF model with an accuracy of 90.6% for CT, 67.1% for mammogram, and 86.9% for MRI. Overall, our detection models showed stronger performance on the CT and mammogram datasets than on the MRI dataset. Out of the studied adversarial detection schemes, the DenseNet and ResNet models showed the best performance, while the Random Forest model showed the poorest ability to identify adversarial images.

Overall, our detection models demonstrate strong performance against attacks with perturbation sizes above a certain threshold. Adversarial attacks with large perturbation sizes that dramatically decrease classification model performance were detected with high accuracy. On the other hand, weaker adversarial attacks with small perturbations were less likely to be detected. That being said, adversarial attacks with smaller perturbations are less likely to cause substantial changes to model classification. The perturbation threshold of detectability is heavily dependent on the perturbation size of adversarial images used to train the detection model. When the detection model is trained on adversarial images with very small perturbation sizes, the detection model is better at detecting adversarial attacks with small perturbations. However, when the perturbation sizes of adversarial attacks used to generate training images for the detection model become too small, the detection model does not train well because the differences in the features of adversarial and normal images become too miniscule for the detector to learn. Our detection schemes are strong in detecting adversarial attacks that pose powerful threats to DL classification models, as those attacks require a certain perturbation size to be effective. 

When exploring the relationship between adversarial detection, adversarial training, or a combination approach on classification accuracy, we found that all three approaches significantly improved classification performance (Table 2). With adversarial images generated with BIM with a fixed perturbation size of 0.004, adversarial detection improved the classification accuracy from 50.58% to 75.63% for CT, from 50.18% to 76.43% for mammogram, and from 50.00% to 74.07% for MRI. Adversarial training improved the classification accuracy to 75.76% for CT, to 66.61% for mammogram, and to 87.88% for MRI. The combined approach improved the classification accuracy to 77.59% for CT, to 70.36% for mammogram, and to 79.99% for MRI. 

## 4. Discussion

Deep learning is a potentially powerful and inexpensive alternative or aid to human decision making for image analysis tasks [29,30,31]. However, as DL models are highly sensitive to adversarial attacks, protecting medical DL models against adversarial attacks is necessary for the safe and effective clinical implementation of DL models. In this study, we compared adversarial detection approaches to differentiate adversarial images from clean images. We found that adversarial attacks with perturbation sizes above a certain threshold can be detected with high accuracy using our detector models. 

Previous studies that have found that adversarial images are highly dangerous to DL models for medical images, dramatically decreasing model accuracy [9,16,32,33]. We extended these findings by investigating the impact of generation methods and varying perturbation sizes of adversarial images on their efficacy at deceiving DL models for medical images [18]. We demonstrated that not all attacks are alike: PGD and BIM attacks are more effective than FGSM attacks, and adversarial images with greater perturbation sizes are more powerful than those with smaller perturbation sizes [18]. In this study, we showed that stronger adversarial images with larger perturbation sizes and a greater impact on classification model performance can be detected with a higher accuracy than adversarial images with smaller perturbation sizes across all detection schemes. 

Our study supports several works which have shown that it is feasible to develop strong approaches to detect adversarial images against DL models for medical images [7,16,33,34,35,36,37]. For example, Li et al. developed an effective unsupervised learning approach using a uni-modal multi-variate Gaussian model (MGM) to detect adversarial images on a deep learning model for chest X-rays [7]. Ma et al. used random forest, SVM, and logistic regression classifiers as detectors for deep features extracted by a neural network, finding high detection accuracy for each method given fixed settings for the adversarial images to be detected [32]. Our work extended this finding by comparing the performance of five detection models against adversarial images, finding some of these—ResNet and DenseNet—to be more consistently robust than others, such as the Random Forest classifier, on DenseNet-extracted features. These results show that detection model architecture is a key determinant of detection success. The detectability of adversarial medical images demonstrates underlying differences between properties of adversarial and clean medical images, as deep features of adversarial images are almost linearly separable from deep features of clean images when 2D embeddings of deep features are visualized using t-SNE [32]. In contrast with non-medical images, deep features for adversarial images closely resemble those for clean images [38,39]. Thus, medical adversarial images are easier to detect than non-medical adversarial images, even though DL models for medical images are more vulnerable to adversarial images than DL models for non-medical images [32].

To our knowledge, our work is the first to compare the effectiveness of adversarial detection, adversarial training, and the combination of adversarial detection and adversarial training to improve classification accuracy. We demonstrated that adversarial training and adversarial detection have comparable effectiveness. There are situations where one approach is superior to the other and vice versa. Furthermore, the use of adversarial training in addition to adversarial detection results in a classification performance that is intermediate to that of either approach alone. Thus, it might be helpful to use a combined approach to optimize classification performance for cases when one particular approach may be weak. This finding can be an important consideration when deciding how to best build robust image classification models for diagnostic use in clinical settings.

Unlike previous studies on adversarial imaging attacks on medical images, we found that our detection schemes underperformed when attempting to identify adversarial images with very small perturbation sizes [7,16,34]. The common limitation in many previous studies investigating adversarial detection for medical images was that they used adversarial images with a constant fixed perturbation size to evaluate the efficacy of the adversarial detector model. However, Shi et al. used an SVM classifier to detect adversarial images using chest x-ray and color fundus datasets and determined the maximum adversarial perturbations their model and human specialists cold detect, finding that detection models greatly outperformed human experts [35]. In our study, we investigated the relationship between varying adversarial perturbation sizes for adversarial images and detection performance, finding that adversarial perturbation size is positively correlated to detector model performance accuracy. Thus, while some adversarial images are more powerful and capable of wreaking havoc on the DL model, they are also more easily detectable. Adversarial images with very small perturbation sizes can fall through the cracks of standard detection schemes, but they are also less effective at decreasing DL model performance. 

Our study has several limitations. First, we only tested on one classification model (VGG16), so our findings may not be applicable to other models. Additionally, some evidence suggests non-convolutional network-based models such as vision transformers maybe more robust to adversarial attacks [33]. Regardless, the VGG16 model shares behavioral similarities with other DL models which comprises a majority of clinically-employed models for image classification, so the findings from this work can be helpful to future works employing other models [40,41,42]. Additionally, some evidence suggests non-convolutional network-based models such as vision transformers maybe more robust to adversarial attacks. Second, our approach only employs white-box attacks where the attack has prior knowledge of access to parameters. It would be helpful to extend the study to black-box attacks, where the attacker cannot see the model parameters, as black-box attacks may be common in real-world settings. Third, we used only first-order adversarial attacks to generate adversarial images, when higher-order attacks exist. Thus, there is a need to investigate the detectability of higher-order adversarial attacks on medical images. 

While exciting progress has been made in the development of adversarial defenses, there is an arms race between the generation of novel adversarial defenses and the creation of adversarial image generation methods that circumvent these defenses [9]. We demonstrate that existing defenses against adversarial images, adversarial detection, and adversarial training cannot fully mitigate the impact of adversarial images against DL models for medical imaging classification. In the current state of DL models, the use of DL-based medical imaging algorithms should be heavily supervised by human clinicians to ensure protection against malicious interventions. Addressing the vulnerability of DL models against adversarial images should be prioritized to fully embrace widespread clinical implementation of DL systems in healthcare systems. Thus, further research into adversarial defense techniques and their effectiveness against medical adversarial images is essential.

## 5. Conclusions

In this work, we applied five different DL-based and ML-based adversarial detection models to compare their effectiveness at differentiating adversarial images from normal images in clinical oncology. We evaluated the performance of our detectors on three cancer imaging datasets of different diagnostic imaging modalities (CT, mammography, and MRI), finding that our detectors exhibit a high detection accuracy for adversarial images with perturbation sizes beyond a certain threshold. Our detection models can discern the adversarial images with larger perturbation sizes capable of dramatically decreasing DL classification model performance. We also demonstrated that the combination of adversarial detection and adversarial training may be a more secure method than the employment of either approach alone. However, we show that neither adversarial detection nor adversarial training can provide universal protection against adversarial images. Thus, future work should focus on detection methods capable of detecting adversarial images with a wider range of perturbation sizes. We believe that our work will facilitate the development of more robust adversarial image detection methods to defend medical deep learning models against adversarial images.

## Figures and Tables

**Table 1:** Accuracy score (%) of DenseNet, Logistic Regression, Random Forest, ResNet, and Support Vector Machine detector models on a combination of normal samples and adversarial samples crafted by designated attack (FGSM, PGD, or BIM) at a set L_∞_ maximum perturbation of 0.004 or 0.008 for lung CT, mammogram, and brain MRI datasets.

		Detection Accuracy (%)					
		FGSM		PGD		BIM	
		ε = 0.004	ε = 0.008	ε = 0.004	ε = 0.008	ε = 0.004	ε = 0.008
CT	DenseNet	99.0	99.1	99.7	99.8	98.4	99.1
	Logistic Regression	95.1	96.7	94.1	96.9	87.6	92.3
	Random Forest	93.9	96.2	90.6	95.8	81.2	89.1
	ResNet	99.3	99.3	100.0	100.0	99.2	99.3
	SVM	93.5	96.0	92.6	96.2	86.9	91.4
Mammogram	DenseNet	99.7	100.0	99.9	100.0	98.7	100.0
	Logistic Regression	70.4	83.8	75.6	84.2	69.8	83.0
	Random Forest	58.8	67.7	67.1	78.9	61.7	75.9
	ResNet	100.0	100.0	100.0	100.0	100.0	100.0
	SVM	67.9	81.0	74.4	82.0	68.7	80.6
MRI	DenseNet	90.0	94.4	80.5	93.5	75.8	91.7
	Logistic Regression	95.1	95.3	93.9	95.3	87.0	95.3
	Random Forest	96.5	97.9	86.9	97.9	70.9	97.9
	ResNet	73.3	89.1	90.0	92.2	85.8	88.4
	SVM	84.1	84.1	83.9	84.1	81.3	84.1

**Table 2:** Accuracy score (%) of classification DL models after application of adversarial detection, adversarial training, or the combination of adversarial detection and adversarial training. The classification model was evaluated on a combination test set of normal and adversarial images. Adversarial images were generated with BIM with a fixed perturbation size of 0.004. For adversarial detection, the ResNet detector was used.

	Classification Accuracy (%)			
	Baseline	Adv Detection	Adv Training	Adv Detection + Training
LIDC	50.58	75.63	75.76	77.59
Mammogram	50.18	76.43	66.61	70.36
MRI	50.00	74.07	87.88	79.99

## Data Availability

Data available upon reasonable request from authors.

## Supplementary Materials

The following supporting information can be downloaded at: https://www.mdpi.com/article/10.3390/cancers15051548/s1, Table S1: Model parameters of VGG16 model used for classification; Table S2: Training parameters for VGG16 classification models for all datasets; Table S3: Model Parameters of ResNet model used for detection; Table S4: Model Parameters of DenseNet model used for detection; Table S5: Model Training Parameters for Detection Models; Table S6: Equations and parameters for FGSM, PGD, and BIM attack methods.
