# Supplementary material for: Comparing Detection Schemes for Adversarial Images against Deep Learning Models for Cancer Imaging
Source: Cancers (Basel). 2023 Mar 1;15(5):1548. doi: 10.3390/cancers15051548 (PMC10000732; doi:10.3390/cancers15051548)
Supplement: Supplementary file 1 [file cancers-15-01548-s001.zip › cancers-2231780-supplementary.pdf]

Supplement

Classification Model Architecture

Table S1. Model parameters of VGG16 model used for classification

Layer (type)	Output Shape	Param #
input_1 (Input Layer)	(None, 224, 224, 3)	0
block1_conv1 (Conv2D)	(None, 224, 224, 64)	1792
block1_conv2 (Conv2D)	(None, 224, 224, 64)	36928
block1_pool (MaxPooling2D)	(None, 112, 112, 64)	0
block2_conv1 (Conv2D)	(None, 112, 112, 128)	73856
block2_conv2 (Conv2D)	(None, 112, 112, 128)	147584
block2_pool (MaxPooling2D)	(None, 56, 56, 128)	0
block3_conv1 (Conv2D)	(None, 56, 56, 256)	295168
block3_conv2 (Conv2D)	(None, 56, 56, 256)	590080
block3_conv3 (Conv2D)	(None, 56, 56, 256)	590080
block3_pool (MaxPooling2D)	(None, 28, 28, 256)	0
block4_conv1 (Conv2D)	(None, 28, 28, 512)	1180160
block4_conv2 (Conv2D)	(None, 28, 28, 512)	2359808
block4_conv3 (Conv2D)	(None, 28, 28, 512)	2359808
block4_pool (MaxPooling2D)	(None, 14, 14, 512)	0
block5_conv1 (Conv2D)	(None, 14, 14, 512)	2359808
block5_conv2 (Conv2D)	(None, 14, 14, 512)	2359808
block5_conv3 (Conv2D)	(None, 14, 14, 512)	2359808
block5_pool (MaxPooling2D)	(None, 7, 7, 512)	0
dropout (Dropout)	(None, 7, 7, 512)	0
flatten (Flatten)	(None, 25088)	0
fc1 (Dense)	(None, 4096)	102764544
dropout_1 (Dropout)	(None, 4096)	0

fc2 (Dense)	(None, 1024)	4195328
predictions (Dense)	(None, 2)	2050
Total params: 121,676,610		

Classification Model Training Parameters

Table S2. Training parameters for VGG16 classification models for all datasets

Parameters	CNN-1 (CT)	CNN-2 (Mammogram)	CNN-3 (MRI)	CNN-4 (MNIST)	CNN-5 (CIFAR-10)
Input Image Size	224 x 224	116 x 116	224 x 224	32 x 32	32 x 32
Batch Size	50	50	50	64	128
Max Epochs	200	100	100	20	60
Patience	50	20	5	0	10
Initial Learning Rate	2e-4	2e-4	2e-4	0.01	0.001
Learning rate decay	1e-6	1e-6	1e-6	0	1e-5
Momentum	0.9	0.9	0.9	0	0.9
Loss	Binary cross-entropy	Binary cross-entropy	Binary cross-entropy	Categorical cross-entropy	Categorical cross-entropy
Optimizer	SGD	SGD	SGD	SGD	SGD

Detection Model Architecture

For the ResNet detection model, we used an ImageNet pretrained ResNet50 as the base network, followed by a global average pooling layer, a dropout layer of rate 0.5, a dense layer of 100 neurons, another dropout layer of rate 0.5, and a 2-neuron dense layer for classification.

Table S3. Model Parameters of ResNet model used for detection

Layer (type)	Output Shape	Param #
resnet50 (Model)	(None, 4, 4, 2048)	23587712
global_average_pooling2d_1	(None, 2048) 0	
dropout_2 (Dropout)	(None, 2048)	0
dense_3 (Dense)	(None, 100)	204900
dropout_3 (Dropout)	(None, 100)	0
dense_4 (Dense)	(None, 2)	202
Total params: 23,792,814		
Trainable params: 23,739,694		
Non-trainable params: 53,120		

For the DenseNet model, we used an ImageNet pretrained DenseNet121 as the base network, followed by a global average pooling layer, batch normalization layer, dropout layer of rate 0.5, a dense layer of 1024 neurons, a dense layer of 512 layers, a batch normalization layer, a dropout layer of 0.5, and a 2-neuron dense layer for classification.

Table S4. Model Parameters of DenseNet model used for detection

Layer (type)	Output Shape	Param #
DenseNet121 (Model)	(None, 3, 3, 1024)	7037504
global_average_pooling2d_2	(None, 1024)	0
batch_normalization_2	(None, 1024)	4096
dropout_4 (Dropout)	(None, 1024)	0
dense_5 (Dense)	(None, 1024)	1049600
dense_6 (Dense)	(None, 512)	524800
batch_normalization_3	(None, 512)	2048
dropout_5 (Dropout)	(None, 512)	0
dense_7 (Dense)	(None, 2)	1026
Total params: 8,619,074		
Trainable params: 8,532,354		
Non-trainable params: 86,720		

For the other three detection models, we first used an ImageNet pretrained DenseNet-121 model to extract deep features from images and separately used support vector machine (SVM), random forest (RF), and logistic regression (LR) as the detection classifiers based on the extracted deep features.

Table S5. Model Training Parameters for Detection Models

Input Image Size	CT	[116, 116, 3]
	Mammogram	[116, 116, 3]
	MRI	[224, 224, 3]
Model	ResNet	DenseNet
Batch Size	64	64
Max Epochs	30	30
Learning Rate	1 e-6	1 e-7
Loss	Binary cross-entropy	Binary cross-entropy
Optimizer	SGD	SGD

Adversarial Attack Methods

Table S6. Equations and parameters for FGSM, PGD, and BIM attack methods. The number of perturbation steps for BIM and PGD are both set to 10, and the step sizes are set to ε/10 and ε/4 for BIM and PGD, respectively.

Attack Type	Equation	Parameters
Fast Gradient Sign Method (FGSM)	$x_{adv} = x + \varepsilon\, sign(\nabla_x J(x,y))$	x_{adv} = adversarial image<br>x = clean input image<br>ε = perturbation size<br>J = loss function<br>y = target label
Projected Gradient Descent (PGD)	$x^0 = x,$<br>$x^t = Clip_{x,\epsilon}\{x^{t-1} + \alpha\, sign(\nabla_x J(x^t,y))\}$	x^0 = clean input image<br>x^i = adversarial image at i^{th}step<br>ε = maximum perturbation size<br>α = perturbation step size<br>J = loss function<br>y = target label<br>Clip{ } function limits updated adversarial sample to within range of ϵ ball ([x-ϵ, x+ϵ]) and the input space ([0,1] for pixel values)
Basic Iterative Method (BIM)	$x^0 = x,$<br>$x^t = \prod_\epsilon (x^{t-1} + \alpha\, sign(\nabla_x J(x^t,y))$	x^0 = clean input image<br>x^i = adversarial image at i^{th}step<br>ε = maximum perturbation size<br>α = perturbation step size<br>J = loss function<br>y = target label<br>y = target label<br>∏ { } function projects the adversarial example back onto the ϵ ball ([x-ϵ, x+ϵ])
